# Tissue metabolic profiling of human gastric cancer assessed by ^1^H NMR

**DOI:** 10.1186/s12885-016-2356-4

**Published:** 2016-06-29

**Authors:** Huijuan Wang, Hailong Zhang, Pengchi Deng, Chunqi Liu, Dandan Li, Hui Jie, Hu Zhang, Zongguang Zhou, Ying-Lan Zhao

**Affiliations:** College of Medicine, Henan University, Kaifeng, 475004 Henan China; State Key Laboratory of Biotherapy and Cancer Center, West China Hospital, West China Medical School, Sichuan University, and Collaborative Innovation Center for Biotherapy, Chengdu, Sichuan People’s Republic of China; Analytical & Testing Center, Sichuan University, Chengdu, 610041 China; Department of Gastrointestinal surgery, West China Hospital, West China Medical School, Sichuan University, Chengdu, 610041 China; Department of Gastroenterology, West China Hospital, West China Medical School, Sichuan University, Chengdu, 610041 China

**Keywords:** Gastric cancer, Tissue, Metabolic profiling, ^1^H-NMR

## Abstract

**Background:**

Gastric cancer is the fourth most common cancer and the second most deadly cancer worldwide. Study on molecular mechanisms of carcinogenesis will play a significant role in diagnosing and treating gastric cancer. Metabolic profiling may offer the opportunity to understand the molecular mechanism of carcinogenesis and help to identify the potential biomarkers for the early diagnosis of gastric cancer.

**Methods:**

In this study, we reported the metabolic profiling of tissue samples on a large cohort of human gastric cancer subjects (*n* = 125) and normal controls (*n* = 54) based on ^1^H nuclear magnetic resonance (^1^H NMR) together with multivariate statistical analyses (PCA, PLS-DA, OPLS-DA and ROC curve).

**Results:**

The OPLS-DA model showed adequate discrimination between cancer tissues and normal controls, and meanwhile, the model excellently discriminated the stage-related of tissue samples (stage I, 30; stage II, 46; stage III, 37; stage IV, 12) and normal controls. A total of 48 endogenous distinguishing metabolites (VIP > 1 and *p* < 0.05) were identified, 13 of which were changed with the progression of gastric cancer. These modified metabolites revealed disturbance of glycolysis, glutaminolysis, TCA, amino acids and choline metabolism, which were correlated with the occurrence and development of human gastric cancer. The receiver operating characteristic diagnostic AUC of OPLS-DA model between cancer tissues and normal controls was 0.945. And the ROC curves among different stages cancer subjects and normal controls were gradually improved, the corresponding AUC values were 0.952, 0.994, 0.998 and 0.999, demonstrating the robust diagnostic power of this metabolic profiling approach.

**Conclusion:**

As far as we know, the present study firstly identified the differential metabolites in various stages of gastric cancer tissues. And the AUC values were relatively high. So these results suggest that the metabolic profiling of gastric cancer tissues has great potential in detecting this disease and helping to understand its underlying metabolic mechanisms.

**Electronic supplementary material:**

The online version of this article (doi:10.1186/s12885-016-2356-4) contains supplementary material, which is available to authorized users.

## Background

Gastric cancer is the fourth most common cancer and the second most common cause of cancer-related death worldwide [1, 2]; it is particularly prevalent in Asian countries, such as China [3, 4]. At present, no effective treatment is available for this disease, and identification of early stage gastric cancer is difficult because of its relatively asymptomatic nature in the early stage and the lack of adequate screening methods. So many patients with gastric cancer are diagnosed at an advanced stage, and they have a high rate of recurrence after resection and a poor survival rate [5, 6]. The 5 years survival rate for early gastric cancer confined to the mucosal or submucosal layer is above 90 % after surgical management [7, 8], yet the 5 years survival rate for advanced gastric cancer is just less than 10 %. Currently, endoscopy is widely used for early screening [9], but this methodology involves invasive procedures and its cost remains disputable. Despite its inconsistent diagnostic efficiency, this stems from variations in the skill and experience of the endoscopist and pathologist. To identify the biomarkers at the early diagnosis of human gastric cancer and improve the survival rate of gastric cancer, efforts have been focused on the identification of patients with poor prognosis and new therapeutic modalities based on molecular mechanisms [10].

Metabolomics, which is the end point of the “-omics” cascade and therefore the last step before phenotype, has been a recently developed technology for the detection, identification and quantification of low molecular weight metabolites that are involved in the metabolism of an organism at a specified time under specific environmental conditions [11, 12]. Recent technological advances in nuclear magnetic resonance (NMR) spectroscopy and mass spectrometry (MS) have also further improved the sensitivity and spectral resolution for cancer metabolic study [13]. Especially NMR has some advantages over MS for metabolic application, including non-destructive analysis, the relative ease of sample preparation, the potential to identify a broad range of compounds and the capacity for the supply of structural information for unknown compounds [14, 15]. In recent years, metabolomics has been used to characterize the metabolic perturbation and identify potential biomarkers in various cancers, such as lung cancer [16], renal cancer [17], colorectal cancer [18]. To our knowledge, only a few reports on metabolic profiling of gastric cancer tissue have been published, and these reports only involved a few patients [19], which cannot provide accurate and comprehensive information of gastric cancer metabolites. Moreover, none of the reports systematically investigated the discriminating metabolites that involved in the different pathological stages of gastric cancer. Therefore, performing metabolic profiling between the different stages of cancer tissues and normal controls will be valuable in aiding diagnosis and understanding of the molecular mechanism involved.

In the present study, we applied ^1^H-NMR to profile the human gastric cancer tissues and normal controls. The metabolic alterations were characterized by orthogonal partial least-squares discriminant analysis (OPLS-DA). On the basis of results, we identified a total of 48 differential metabolites. These modified metabolites potentially revealed disturbance of energy, amino acids, ketone body and choline metabolism in human gastric cancer. We also intended to gain knowledge of potential metabolic biomarkers associated with gastric cancer, which can be used for early diagnosis, staging and therapeutic strategies.

## Methods

### Sample collection and chemical regents

125 gastric cancer patients were recruited during 2012 to 2013, a total of 179 surgical specimens were collected. Among them, 108 cases belonged to the matched tumor and normal control, which were taken at least 5–10 cm away from the edge of a tumor from the same patient (*n* = 54). The tissues dissected by a senior pathologist in the operating room were immediately frozen in liquid nitrogen and stored at −80 °C.

The patients enrolled in this study did not receive any neoadjuvant chemotherapy or radiation therapy before surgical treatment. The pathological diagnosis was confirmed in routine histopathological H & E stained specimens and categorized according to postoperative classification of malignant tumors (TNM): stage I, 30 patients; stage II, 46 patients; stage III, 37 patients; stage IV, 12 patients.

Deuterium water (99.8 % D) was purchased from CIL (Cambridge Isotope Laboratories, USA). Trimethylsilylpropionic acid-d4 sodium salt (TSP) was purchased from Sigma Aldrich (USA). HPLC-grade methanol was purchased from Fisher Scientific (USA). HPLC-grade chloroform was purchased from Scharlau (Spain). All of the other chemicals employed in this study were of analytic pure and culture grade.

### Sample preparation for NMR analysis

To extract the metabolites of interest (e.g., carbohydrates, lipids, amino acids and other small metabolites), the 150–400 mg of frozen tissue samples were placed into a 1.5 mL eppendorf vials and weighed. Methanol (4 ml per gram of tissue) and double distilled water (0.85 ml per gram of tissue) were added and the mixtures were vortexed for 1 min. Chloroform (2 ml per gram of tissue) was then added. The samples were kept on ice for 30 min to extract metabolites, followed by centrifugation at 1000 g for 30 min at 4 °C. This procedure should separated suspension into three phases: the water phase at the top, the denatured proteins phase in the middle, and the lipid phase at the bottom. The upper aqueous phases of each sample were transferred into differently new 1.5 ml eppendorf vials and evaporated to dryness under a stream of nitrogen. The residue was redissolved with 580 μl of D2O, containing 30 μM phosphate buffer solution (PBS, pH = 7.4) and 0.01 mg/ml sodium (3-trimethylsilyl)-2,2,3, 3-tetradeuteriopropionate (TSP), which provided the deuterium lock signal for the NMR spectrometer and the chemical shift reference (δ0.0), respectively. After centrifugation at 12,000 g for 5 min at 4 °C, the 550 μl supernatant was transferred into a 5-mm NMR tube for NMR spectroscopy [20].

### ^1^H-NMR spectroscopic analysis

The ^1^H NMR spectra of all tissue samples were acquired on a Bruker Avance II 600 spectrometer operating (Bruker Biospin, Germany) at 600.13 MHz and a temperature of 300 K. A one-dimensional spectrum was acquired by using a standard (1D) Carr-Purcell-Meiboom-Gill (CPMG) pulse sequence to suppress broad signals from bigger molecules, such as lipids and proteins. Sixty-four free induction decays (FIDs) were collected into 64 K data points with a spectral width of 12,335.5Hz spectral, an acquisition time of 2.66 s, and a total pulse recycle delay of 7.66 s. The FIDs were weighted by a Gaussian function with line broadening factor of 0.3 Hz, Gaussian maximum position 0.1, prior to Fourier transformation [21].

### ^1^H-NMR spectral data processing

To reduce the complexity of the NMR data and facilitate the pattern recognition, the raw NMR data (FIDs) were manually Fourier transformed using MestReNova-6.1.1-6384 software before data processing. The ^1^H NMR spectra of all tissue samples were phase adjusted and baseline corrected after referencing to TSP resonance at δ0.0. The spectra ranging from 9.5 to 0.5 ppm was subsequently divided into 4500 integral segments corresponding to 0.002 ppm. The regions 7.84-7.62 ppm (chloroform),4.94–4.66 ppm (water) and 3.37–3.34 ppm (methanol) were removed. Moreover, the integrated data were normalized before pattern recognition analysis to eliminate the dilution or bulk mass differences among samples due to the different weight of tissue, and to give the same total integration value for each spectra.

### Multivariate statistical analysis

OPLS-DA was performed using standard procedures for multivariate statistical analysis in statistical software SIMCA-P + 11 (Umetrics, AB). To separate the tumor samples from the normal controls, the goodness-of fit parameter (R^2^) and the goodness of prediction parameter (Q^2^) values were used to assess the quality of the models, respectively. The PLS-DA (partial least-squares discriminant analysis) models were cross-validated by a permutation analysis (200 times) [22], and the resulting R^2^ and Q^2^ values were calculated. The default 7-round cross-validation was applied with 1/seventh of the samples being excluded from the mathematical model in each round, in order to guard against overfitting. The *y* variables as specific model coefficients locate the NMR variables. The model coefficients were then back-calculated from the coefficients incorporating the weight of the variables in order to enhance interpretability of the model: in the coefficient plot, the intensity corresponds to the mean-centered model (variance) and the color-scale derives from the unit variance-scaled model (correlation). The coefficient plots were generated with Matlab scripts with some in-house modifications and were color-coded with the absolute value of coefficients (*r*) [23]. The differentiation performance (specificity and sensitivity) was assessed by the area under the curve (AUC) of the receiver operating characteristic (ROC) curves. The ROC analysis was also performed to validate the robustness of the OPLS-DA models using the predicted *Y* values of samples of internal (seven-fold) and external validation sets.

To identify the interesting spectrum peaks between tumor tissues and normal controls, the variable importance in the projection (VIP) values of all peaks from OPLS-DA models were analyzed and taken as a coefficient, and variable with VIP > 1 was considered relevant for group discrimination. Moreover, unpaired Student’s *t*-test (*p* < 0.05) to the chemical shifts was also used to assess the significance of each metabolite. Besides, false- discovery rate (FDR) and adjust *p*-value for multiple testing were also supplied. Only both meeting VIP > 1 and *p* < 0.05, the metabolite was identified as distinguishing one. The corresponding chemical shift and multiplicity of the metabolites were identified by comparisons with the previous literatures and the Human Metabolome Database (http://www.hmdb.ca/).

## Results

### Study population

We investigated a total of 179 tissue samples, 125 of which were gastric cancer tissue (91 males and 34 females; age range, 28–86 years; median age, 60 years), and 54 of which were normal controls (39 males and 15 females; age range, 28–80 years; median age, 61 years). Among them, 108 cases belonged to the matched tumor and normal control from the same patient (*n* = 54). The clinicopathological characteristics of gastric cancer patients were summarized in Table 1. As shown in Table 1, the stage of all tissue specimens was determined according with the American Joint Committee on Cancer (AJCC) for gastric cancer: stage I, 30 patients; stage II, 46 patients; stage III, 37 patients; stage IV, 12 patients. All patients were subjected to surgical resection of the primary tumor and dissection of lymph nodes.

**Table 1:** Clinical information for gastric cancer patients and normal controls analyzed by ^1^H NMR

	Gastricl cancer patients	Normal controls
Number	125	54
Age (median, range)	60 28–86	61 28–80
Male/female ration	91/34	39/15
Histology	Adenocarcinoma (120)	∕
	NA (5)	
Pathologic grade		∕
PD	74	
MD	46	
WD	0	
NA	5	
Cancer stage/Duke		∕
I/A (30)	T1N0M0 (7)	
	T1N1M0 (4)	
	T2N0M0 (19)	
II/B (46)	T2N1M0 (5)	
	T3N0M0 (15)	
	T2N2M0 (1)	
	T3N1M0 (12)	
	T4aN0M0 (13)	
III/C (37)	T2N3M0 (3)	
	T3N2M0 (10)	
	T4aN1M0 (8)	
	T4aN2M0 (9)	
	T4aN3aM0 (1)	
	T4bN1M0 (4)	
	T4bN2M0 (2)	
IV/D (12)	T2N1M1 (1)	
	T3N1M1 (1)	
	T3N2M1 (4)	
	T3N3aM1 (2)	
	T4aN3aM1 (4)	

*PD* poorly differentiated, *MD* moderately differentiated, *WD* well-differentiated, *NA* not applicable

### ^1^H NMR metabolic profiling of sample

**Fig. 1:**
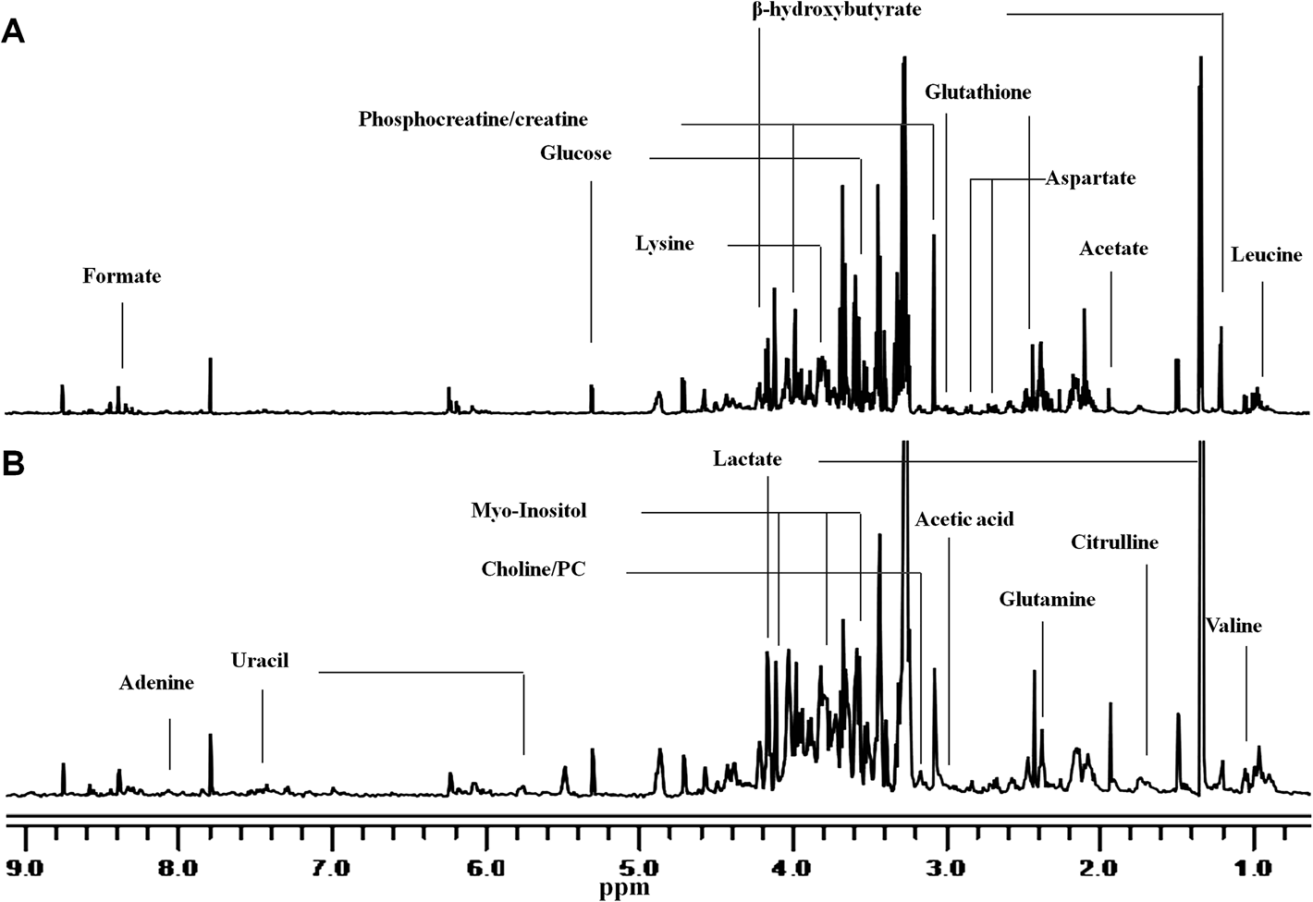
600 MHz representative ^1^H NMR spectra (δ9.5–δ0.5) of tissue samples. **a** means normal control, (**b**) means gastric cancer tissue

We obtained NMR spectrum of the tissue samples from gastric cancer and normal control. The representative ^1^H NMR spectrum of aqueous phase extracts of gastric cancer and normal control were showed (Fig. 1). The standard one-dimension spectrum gave an overview of all metabolites. The major spectrum can be assigned to specific metabolites by comparing their chemical shifts and spectral peak multiplicities with literature data and spectra of standards acquired in Human Metabolome Database (http://www.hmdb.ca/). Inspection of Fig. 1 showed clear visible differences between gastric cancer (Fig. 1b) and normal control (Fig. 1a). As a result, a series of changes of endogenous metabolite levels were observed. The spectral region from 0.5 to 3.0 ppm included some signals, such as leucine, valine, lactate, citrulline, acetate, glutamine, glutathione, aspartate, acetic acid. The region from 3.0 to 5.0 ppm contained many signals, including myo-inositol, choine, PC, lysine, glucose, β-hydroxybutyrate, and so on. The certain signals from 5.0 to 9.5 ppm were few, including glucose, uracil, adenine and formate. These metabolites were known to be involved in multiple biochemical processes, especially in energy and amino acid metabolism [24, 25].

### Multivariate statistical analysis of gastric cancer tissues and normal controls

**Fig. 2:**
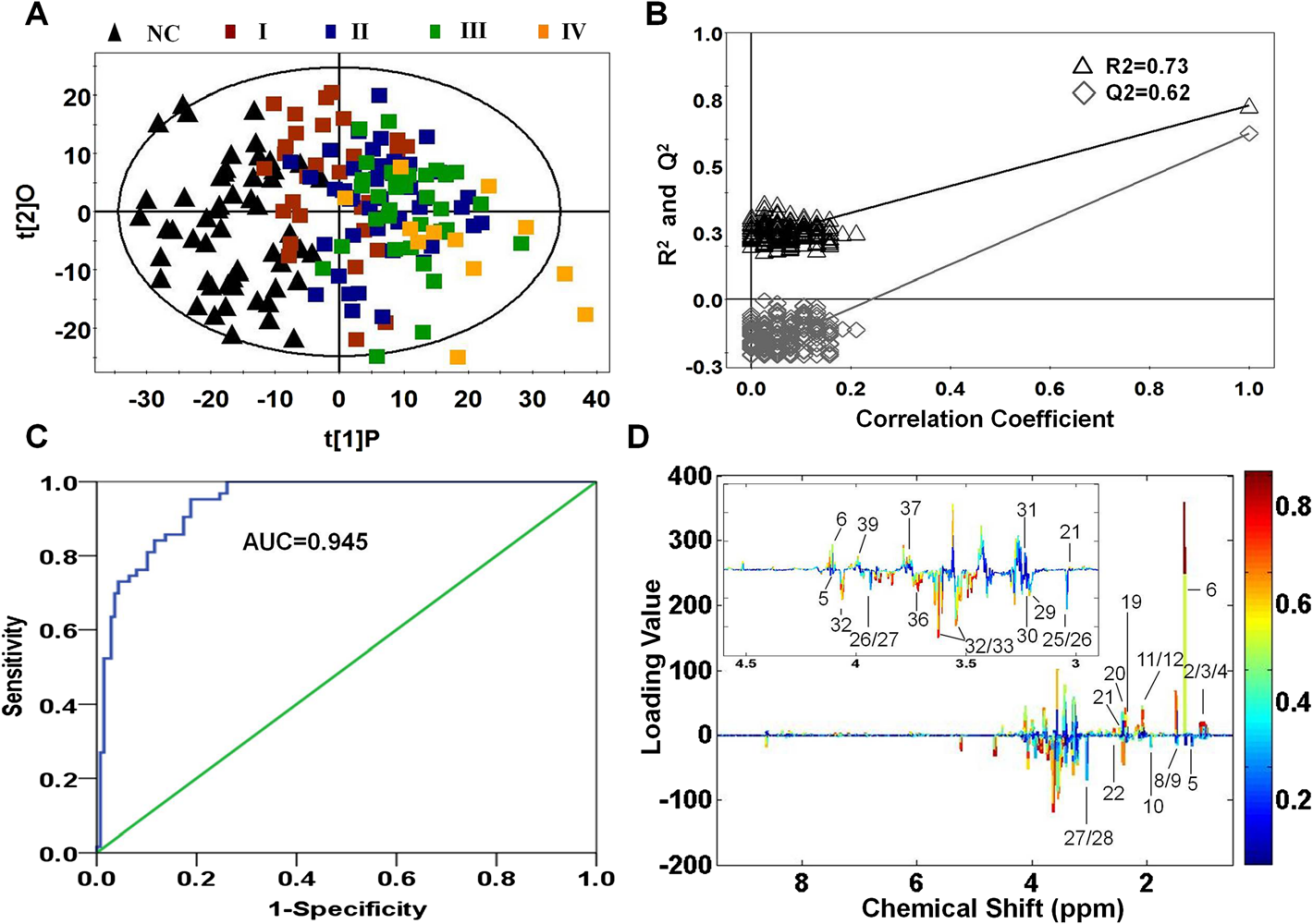
Metabolic profiling between gastric cancer tissues and normal controls. **a** OPLS-DA scores plot between the gastric cancer tissues and normal controls using ^1^H NMR. Black triangles represent normal controls, red blocks represent stage I of gastric cancer tissues, blue blocks represent stage II, green blocks represent stage III, yellow blocks represent stage IV. **b** Statistical validation of the corresponding PLS-DA model using permutation analysis (200 times). R^2^ is the explained variance, and Q^2^ is the predictive ability of the model. **c** ROC analysis was performed using the *Y*-predicted value determined by the OPLS-DA model. AUC value of this OPLS-DA model was 0.945. **d** The color map showed the significance of metabolite variations between the two classes. The color close to blue means the trend of metabolite change was smaller, The color close to red means the trend of metabolite change is bigger. The color value represents the relative degree of metabolite changes. Peaks in the positive direction indicate the increased metabolites in gastric cancer tissues in comparison to normal controls. Peaks in the negative direction indicate the decreased metabolites

First, PCA (principal component analysis) was applied to examine intrinsic variation between gastric cancer tissues and normal controls after ^1^H NMR data normalization. The PCA scores plot showed that cancer group and normal group samples were scattered into different regions (Additional file 1). The majority samples were located in 95 % confidence interval. Therefore, all of samples were used in the following analysis to ensure the maximum information. Next, to enhance the separation of the two groups, OPLS-DA was performed to minimize the possible contribution of intergroup variability. As shown in Fig. 2a, OPLS-DA showed a good separation pattern between gastric cancer tissues (color blocks) and normal controls (black triangles). Moreover, model parameters in the permutation plot for the explained variation (R^2^ = 0.73) and the predictive capability (Q^2^ = 0.62) were significantly high, demonstrating it was an excellent model and showing high predictability values (Fig. 2b).

To validate the robustness of the OPLS-DA model in discriminating cancer tissues from controls, ROC analysis was performed using the predicted *Y* values of samples of internal (seven-fold) and external validation sets based on OPLA-DA model. Area under the curve (AUC) value of this model was 0.945 (Fig. 2c), which showed that the OPLS-DA model gave a good diagnostic value for gastric cancer. Of note, this diagnostic model was just to identify the tissue metabolic biomarkers rather than to replace the established histopathologic diagnostic standard for gastric cancer.

Based on the NMR profiling, we totally identified 56 metabolites between gastric cancer tissues and normal controls. To find out the main metabolites discriminating gastric cancer tissues from normal controls, the metabolites (VIP < 1 or *p* > 0.05) were removed and the significantly distinguishing metabolites according to VIP > 1 and *p* < 0.05 were listed in Table 2. The OPLS-DA loadings were colored according to the absolute value of coefficients (Fig. 2d) and showed the significant class-discriminating metabolites responsible for the clustering patterns. Positive signals, corresponding to the up-regulated metabolites in gastric cancer tissues in comparison to normal controls, were found for isoleucine, leucine, valine, lactate, N-acetyl glycoprotein, O-acetyl glycoprotein, succinate, glutamine, glutathione, TMAO, lysine and serine. On the other hand, the negative signals indicated the down-regulated metabolites in gastric cancer tissue, including β-hydroxybutyrate, citrulline, acetate, methylamine, phosphocreatine, creatine, ceatinine, acetic acid, choline, phosphochline, myo-Inositol, glucose, dimethylglycine.

**Table 2:** Differential Metabolites derived from OPLS-DA model of ^1^H NMR analysis between gastric cancer patients and normal controls

Metabolites	Chemical shift	Mutiplicity^a^	Gastric cancer vs Normal control				
	(ppm)		VIP^b^	FC^c^	*P* Value^d^	Adjust *p* value	FDR
1 VLDL: CH_3_-(CH_2) n-_	0.892	br	1.15	−1.31	0.015	0.003	0.020
2 Isoleucine	0.942	t	2.35	1.12	0.000	0.000	0.000
	1.014	d	3.05	1.37	0.000	0.000	0.000
3 Leucine	0.96	t	2.28	1.02	0.000	0.000	0.000
4 Valine	0.99	d	2.81	1.12	0.000	0.000	0.000
	1.01	d	2.81	1.10	0.000	0.000	0.000
5 β-hydroxybutyrate	1.2	d	1.03	−1.31	0.002	0.007	0.003
	4.16	d	2.27	−1.02	0.000	0.000	0.000
6 Lactate	1.33	d	3.62	1.22	0.000	0.000	0.000
	4.11	q	2.14	1.16	0.000	0.000	0.000
7 2-Hydroxyisobutyric acid	1.44	s	1.26	−1.02	0.000	0.000	0.000
8 Citrulline	1.57	m	2.51	−2.25	0.000	0.000	0.000
9 VLDL: −CH_2_-CH_2_-CH_2_O	1.58	br	2.64	−1.76	0.000	0.000	0.000
10 Acetate	1.93	s	2.24	−1.19	0.000	0.000	0.000
11 N-Acetyl glycoprotein	2.05	s	1.81	1.14	0.000	0.000	0.000
12 O-Acetyl glycoprotein	2.07	s	3.32	2.10	0.000	0.000	0.000
13 D-ribose	2.23	s	1.84	−1.56	0.000	0.000	0.000
14 Acetone	2.23	s	1.84	−1.56	0.000	0.000	0.000
15 Lipid,-CH_2_-C = O	2.26	br	1.87	−1.18	0.000	0.000	0.000
16 Acetoacetate	2.28	s	1.34	−1.16	0.019	0.013	0.027
17 Acetoacetic acid	2.31	s	1.50	−1.30	0.000	0.000	0.000
18 Glutamate	2.356	m	2.65	1.29	0.000	0.000	0.000
	3.768	m	2.52	1.18	0.000	0.000	0.000
19 Succinate	2.41	s	2.66	1.01	0.000	0.000	0.000
20 Glutamine	2.45	m	2.24	1.02	0.000	0.000	0.000
21 Glutathione	2.56	m	2.41	1.51	0.000	0.000	0.000
	2.96	m	2.28	1.45	0.000	0.000	0.000
22 Methylamine	2.59	s	2.03	2.26	0.000	0.000	0.000
23 Aspartate	2.68	dd	1.38	1.54	0.000	0.000	0.000
	2.82	dd	1.62	1.56	0.000	0.000	0.000
24 Dimethylamine	2.732	s	1.43	1.02	0.040	0.045	0.045
25 Acetic acid	3.00	s	1.99	−1.09	0.000	0.000	0.119
26 Phosphocreatine	3.04	s	1.95	−1.08	0.000	0.000	0.000
	3.93	s	1.12	−1.08	0.000	0.000	0.000
27 Creatine	3.04	s	1.95	−1.08	0.000	0.000	0.000
	3.94	s	1.12	−1.08	0.000	0.000	0.000
28 Ceatinine	3.04	s	1.95	−1.08	0.000	0.000	0.000
	3.448	s	1.03	−1.00	0.004	0.017	0.007
29 Choline	3.2	s	1.33	−1.34	0.000	0.011	0.000
30 PC (phosphochline)	3.21	s	1.78	−1.38	0.000	0.000	0.000
31 Trimethylamine-N-oxide (TMAO)	3.27	s	1.67	1.71	0.000	0.000	0.000
32 myo-Inositol	3.54	dd	2.69	−1.68	0.000	0.000	0.000
	3.63	t	2.73	−1.42	0.000	0.000	0.000
	4.06	m	2.71	−1.49	0.000	0.000	0.000
33 α-Glucose	3.54	dd	2.69	−1.68	0.000	0.000	0.000
	5.23	d	2.98	−2.83	0.000	0.000	0.000
34 Glycine	3.57	s	1.89	−1.33	0.056	0.040	0.046
35 Glycerol	3.65	dd	2.27	−1.51	0.000	0.000	0.000
36 Dimethylglycine	3.71	s	2.90	−2.18	0.000	0.000	0.000
37 Lysine	3.77	m	2.52	1.18	0.000	0.000	0.000
38 Glycolate	3.93	s	1.12	−1.08	0.000	0.000	0.000
39 Serine	3.98	m	2.14	1.10	0.000	0.000	0.000
40 Uracil	5.8	d	2.16	4.89	0.000	0.000	0.000
	7.54	d	2.11	2.33	0.000	0.000	0.000
41 Fumarate	6.53	s	1.23	1.20	0.001	0.007	0.002
42 4-hydroxyphenylactate	6.88	d	2.40	1.51	0.000	0.000	0.000
	7.18	d	2.59	1.22	0.000	0.000	0.000
43 Tyrosine	6.9	d	2.40	1.53	0.000	0.000	0.000
	7.2	d	2.59	1.22	0.000	0.000	0.031
44 Trytophan	7.29	m	1.36	−1.39	0.000	0.000	0.000
45 Phenyacetylglutamine	7.42	m	2.55	1.11	0.000	0.000	0.000
46 Adenine	8.12	m	1.26	1.21	0.002	0.000	0.004
47 Hypoxanthine	8.18	s	1.48	−1.08	0.001	0.001	0.003
	8.21	s	1.48	−1.42	0.000	0.001	0.000
48 Formate	8.44	s	1.39	−1.04	0.009	0.009	0.015

^a^Multiplicity: *s* singlet, *d* doublet, *t* triplet, *q* quartet, *dd* doublet of doublets, *m* multiplet, *br* broad; ^b^Variable importance in the projection was obtained from OPLS-DA model with a threshold of 1.0. ^c^Fold change (FC) between gastric cancer patients and normal controls. Fold change with a positive value indicates a relatively higher concentration present in gastric cancer patients while a negative value means a relatively lower concentration as compared to the normal controls. ^d^ *P*-value obtained from Student’s *t*-test

### Multivariate statistical analysis between stage-related gastric cancer tissues and normal controls

**Fig. 3:**
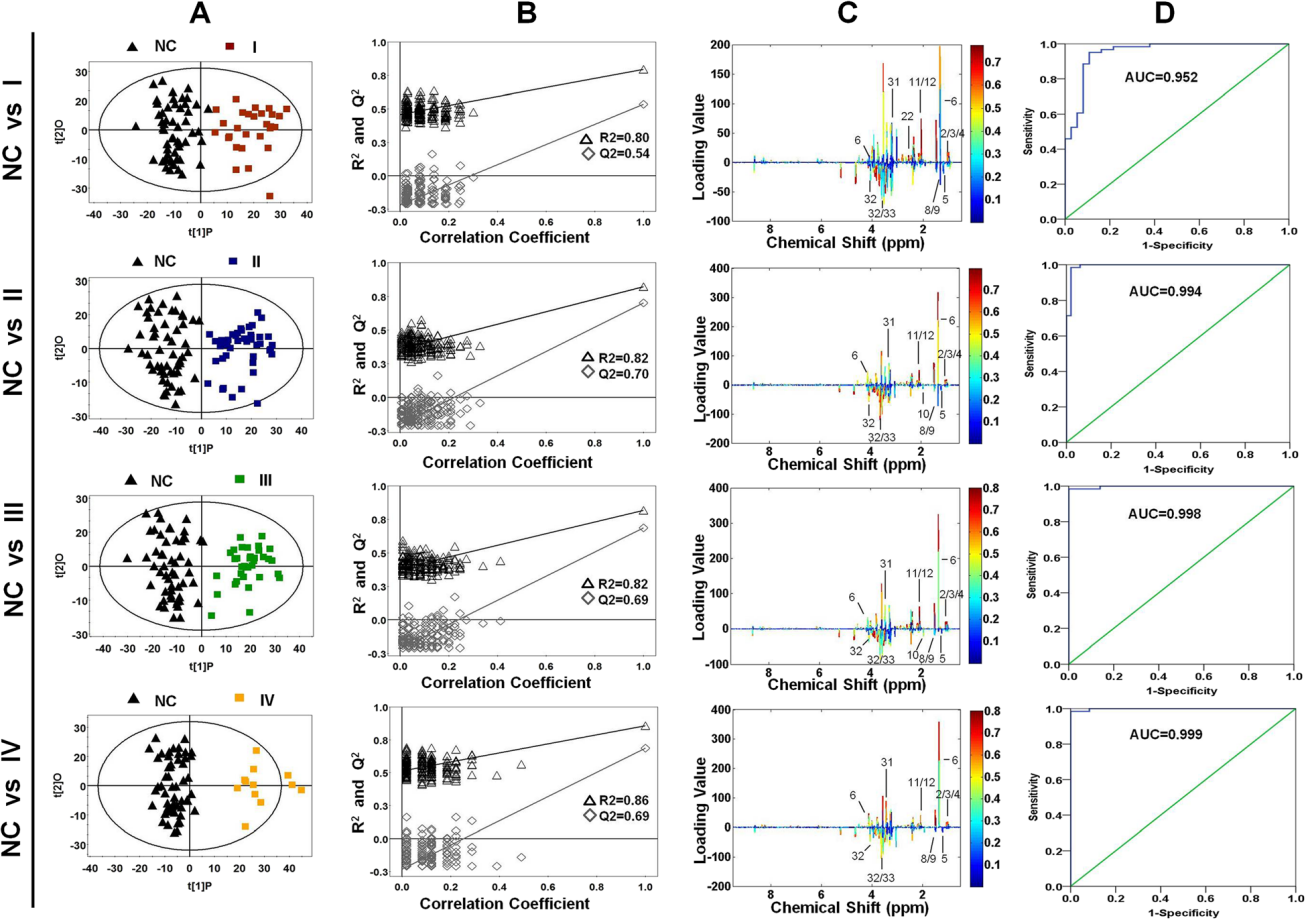
Metabolic profiling between different stages of gastric cancer tissues and normal controls. **a** OPLS-DA scores plots based on each stages of gastric cancer tissues and normal controls. **b** Statistical validation of the corresponding PLS-DA models using permutation analysis (200 times). R^2^ is the explained variance, and Q^2^ is the predictive ability of the model. **c** Color map showed the significance of metabolite variations between the classes. Peaks in the positive direction indicated the increased metabolites in gastric cancer tissues. Decreased metabolites in gastric cancer tissues were presented as peaks in the negative direction. **d** ROC analysis was performed using the *Y*-predicted value determined by the OPLS-DA model between the classes

Performing metabolic profiling between various stages of gastric cancer and normal controls will be valuable in aiding accurate diagnosis and therapy and understanding of the molecular mechanism involved. To our knowledge, this study was the first to show the differences of metabolic profiling among various stages of gastric cancer. According to the multivariate statistical analysis of gastric cancer tissues and normal controls, many distinguishing metabolites have been found. Similarly, OPLS-DA model was applied to analyze the metabolic difference between each stages of gastric cancer and normal controls. As shown in Fig. 3a, the score plots showed that all stages (I, II, III, IV) of gastric cancer tissues could be clearly separated from normal controls. And there was also a trend of separation among different stages (Additional files 2 and 3). A total of 48 distinguishing metabolites with VIP > 1 from the training set and *p* < 0.05 from Student’s *t*-test were identified and summarized in Additional file 4. The majority were similar to those of metabolites between gastric cancer and normal controls. As shown in Additional file 4, the VIP values of isoleucine, lactate, glutamate, glutathione, TMAO, 4-hydroxyphenylactate, tyrosine, phenyacetylglutamine and hypoxanthine were increased along with the progression of the gastric cancer, which indicated these metabolites played increasingly important role in separation stage-related gastric cancer tissue. The FC (fold change) of citrulline, valine, and acetoacetate were increasingly changed from stage I to stage IV, suggesting the expression of these metabolites were growing along with the progression of gastric cancer. However, the FC of methylamine was decreased, especially in stage IV. Totally, the change of these metabolites indicated that they would play an important role in the progression of disease, the underlying mechanism may need more future work.

The permutation analysis of the corresponding OPLS-DA, were shown in Fig. 3b. The parameters for different stages were as follows: stage I: R^2^ = 0.80, Q^2^ = 0.54; stage II: R^2^ = 0.82, Q^2^ = 0.70; stage III: R^2^ = 0.82, Q^2^ = 0.69 and stage IV: R2 = 0.86, Q2 = 0.69, which indicated the excellence of the model. To get an insight into the types of metabolites responsible for the separation between different subjects, the corresponding loading plots based on OPLS-DA models were presented in Fig. 3c. The relative changes in metabolites with significant correlation coefficients were a major discriminating factor among different subjects, implying the biochemical alterations in different morbidity. ROC analysis was performed to detect the predictive power of OPLS-DA model. As shown in Fig. 3d, the corresponding AUC values were 0.952, 0.994, 0.998 and 0.999, indicating the OPLS-DA model exhibited a good diagnostic value for gastric cancer.

## Discussion

In the present study, we discriminated the metabolic profiling of 125 gastric cancer tissues from 54 normal controls based on ^1^H NMR, and analyzed the metabolic difference between the each stage of gastric cancer and normal controls to identify the potential biomarkers involved in the progression of gastric cancer. A total of 48 distinguishing metabolites were identified and 13 of them were changed along with the development of gastric cancer, including isoleucine, lactate, glutamate, glutathione, TMAO, 4-hydroxyphenylactate, tyrosine, phenyacetylglutamine, hypoxanthine, citrulline, valine, acetoacetate and methylamine. Compared with the published reports of the metabolic profiling of gastric cancer tissues [19], the present study identified more distinguishing metabolites, which 48 metabolites were contrast with 12 and 18 metabolites. The large cohort of tissue samples (179 subjects) may be an important reason for the more identified metabolites. More importantly, to the best of our knowledge, the present study was the first to demonstrate the metabolic difference between the various stages of gastric cancer and normal controls, which will be valuable in aiding accurate diagnosis and understanding of the potential molecular mechanism.

**Fig. 4:**
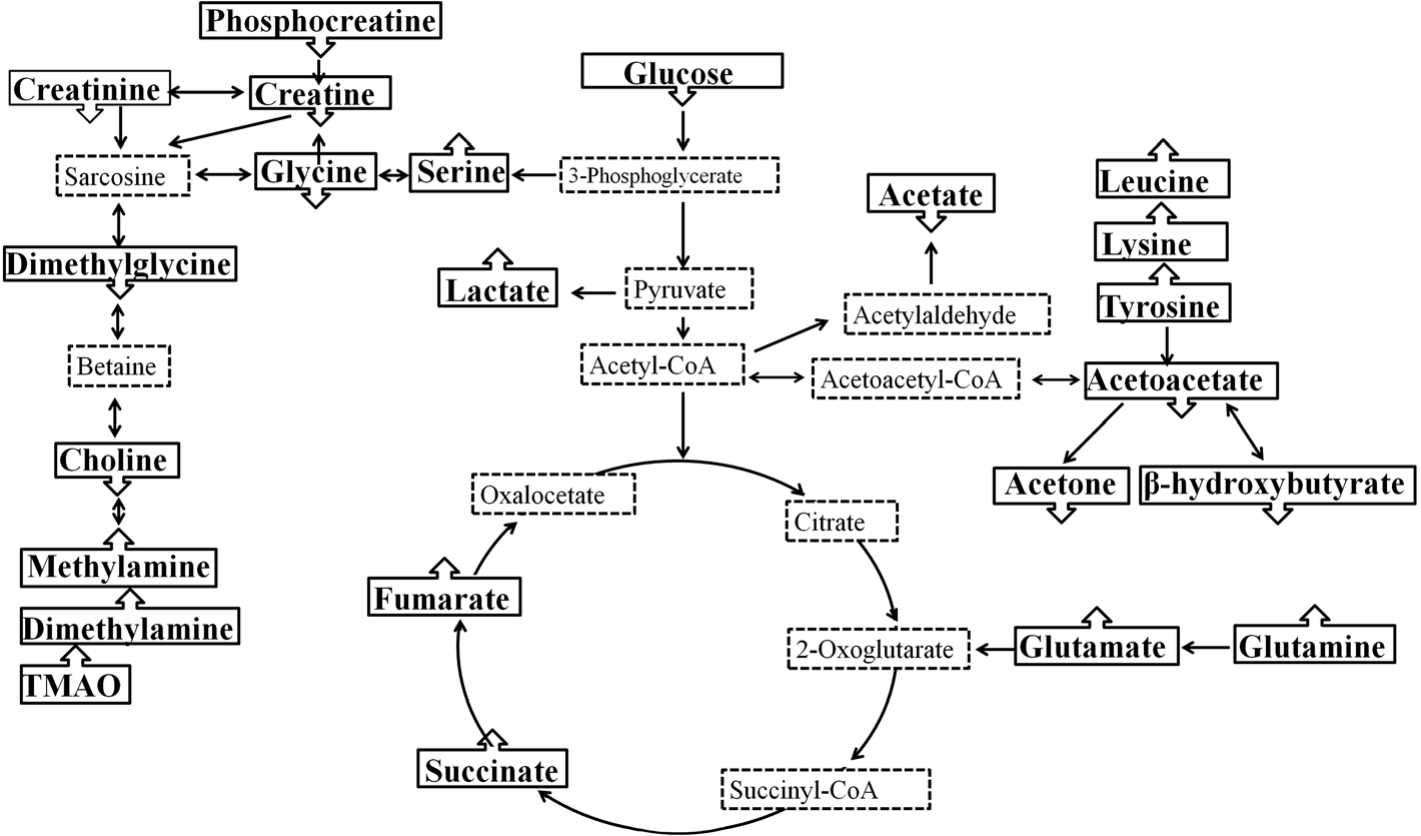
Metabolic pathway of significantly changed metabolites between gastric cancers and normal controls. The up arrows represent the metabolites increased in the gastric cancer tissues in comparison to normal controls. The down arrows represent the metabolites decreased in the gastric cancer tissues. Dashed lines surrounding compounds mean not measured or not significant between two groups

To understanding the possible connections among these tissue metabolites, we constructed the related metabolic pathway maps based on the modified metabolites and information obtained from the Kyoto Encyclopedia of Genes and Genomes Web site (www.genome.jp/kegg/), which was shown in Fig. 4, and the relative changes between gastric cancer tissues and normal controls was shown in Additional file 5. The disturbed metabolic pathway included glycolysis (glucose and lactate), tricarboxylic acid cycle (TCA) (succinate and fumarate), glutaminolysis (glutamine and glutamate), serine synthesis (serine and glycine), ketoplasia (acetoacetate, β-hydroxybutyrate and acetone), choline metabolism (TMAO, dimethylamine, methylamine, choline and dimethylglycine) and amine acid metabolism (leucine, lysine, tyrosine, serine and glycine).

As shown in Fig. 4 and Additional file 5, mean glucose levels from gastric cancer tissues were significantly lower than in normal controls. Meanwhile, mean lactate levels were increased in gastric cancer tissues, which matched previous reports [26, 27]. The results were not surprised because of the well-known Warburg effect [28, 29]. Increased glycolysis is proposed to be associated with many tumors and with cancer cell growth, cancer cells prefer to utilize 1 molecule glucose through glycolysis to generate 2 molecules ATP instead of 36 molecules ATP through oxidative phosphorylation even in presence of ample oxygen. This process is less efficient, so cancer cells must enhance glucose uptake to meet the energy requirement maintaining their quickly growth and proliferation. In gastric cancer cells, the expressions of glucose transporters (Glut-1 and Glut-3) were up-regulated to transport more glucose into cells to satisfy the great amount of energy requirements [30, 31]. Lactate, as the end product of glycolysis, was found to accumulate in gastric cancer tissues along with the decrease of glucose. Lactate is able to make tumor microenvironment consistently acidic, which would stimulate tumor cell metastasis in vivo and invasion in vitro [25, 32]. Pyruvate kinase M2 isoform (PKM2), a key regulator of glycolysis, controls glucose afflux to lactate, which is high expression of many cancers [33]. So knockdown of PKM2 expression will inhibit glycolysis, which may aid in the design of new therapy for the treatment of cancer [34, 35].

The preferential conversion of glucose to lactate in cancer cells is believed one of the metabolic differences between cancer and normal controls. However, the extent to which glucose-derived metabolic fluxes are used for alternative processes is poorly understood. In the present study, a higher level of serine in gastric cancer tissues was observed, so the serine synthesis pathway (SSP) was activated, which regulated the intracellular synthesis of serine and glycine. Under the metabolic stress, cancer cells rapidly used exogenous serine and serine deprivation triggered activation of SSP, which will suppress glycolysis and increase flux to tricarboxylic acid cycle [36]. So the utility of serine depletion will open a new therapeutic window in cancer cells that show some sensitivity to serine depletion. Moreover, 3-phosphoglycerate dehydrogenase (PHGDH), a key metabolic enzyme of SSP, was reported to amplify in melanoma [37] and triple-negative breast cancer [38]. Reducing PHGDH expression impaired the cancer cell proliferation, whereas overexpression of PHGDH in human breast cancer contributed to carcinogenesis by facilitating glycolysis to SSP [39]. These observations together with our findings strongly supported a hypothesis that altered serine metabolism occur in human gastric cancer.

In mammalian cell, glucose and glutamine are two of the most abundant nutrients to support energy, precursors for macromolecular synthesis, and substrates for other essential functions [40]. However, the oxidative phosphorylation of glucose in the mitochondria is impaired in cancer cells, which is termed Warburg effect. The amount of glucose-derived acetyl-CoA entering into TCA cycle decreases significantly. As a result, cancer cells rely on alternate metabolites to replenish TCA cycle intermediates. So glutaminolysis playes an important role in generating ATP and maintaining the mitochondrial function. Glutamine serves as a major source for energy and nitrogen for biosynthesis, and a carbon substrate for anabolic processes in cancer [41]. As shown in Fig. 4, glutamine is converted to glutamate by glutaminase (GLS), which release the amide nitrogen of glutamine as ammonia. Glutamate is converted to α-ketoglutarate (AKG) by two types of reactions, which enter into TCA to support energy and biosynthesis in mitochondrion. So the alternative modes of metabolism of glucose and glutamine enable cancer cells to resist metabolic stress and contribute to cancer cells survive and growth.

Amino acids play a pivotal role in several metabolic pathways and are highly essential in performing specialized functions inside the cell. In this study, Amino acids that were found to be significantly different between cancer tissues and controls were listed in Table 2 and Additional file 4. In addition to higher levels of glutamine and glutamate, isoleucine, leucine, valine, lysine, serine and tyrosine were increased in gastric cancer tissues. The source of these amino acids has not been determined. Some reports considered that it is likely to be a combination of systemic protein catabolism and the degradation of extracellular matrix [42]. And the others thought it could be attributed to the uptake by cancer cells from normal organ and blood through the up-regulation of amino acid transporters [43, 44]. In a word, amino acids are as the basic unit in protein structure and the precursor for purine and pyrimidine biosynthesis, their disturbances reflect the needs for the rapid proliferation of cancer cells. The level of uracil, as a precursor in ribonucleic acid, was apparently higher in gastric cancer tissues (about 5 fold), which similarly suggested cancer cells were in the state of rapid growth and proliferation.

Citrulline, a naturally non-essential amino acid, was firstly found in watermelon, Apart from its role in protein homeostasis and as an intermediate in urea cycle, citrulline is also found to be a potent hydroxyl radical scavenger and much more effective precursor of arginine and NO than arginine itself [45, 46]. The level of citrulline decreased along with the processes of gastric cancer, which may suggest the deterioration of the redox state of tumor. The potential mechanism needs further exploration.

Choline is an essential nutrient, which plays a critical role in the structure and function of biological membranes in all cells as an essential precursor of cell membrane phospholipids [47]. Choine and betain may act as methyl group sources in folate-mediated one-carbon metabolism, which may affect carcinogenesis by influencing methylation and synthesis of DNA [48]. In the present study, the choline metabolic pathway was disorder. Phosphocreatine, creatine, creatinine, dimethylgcine and choline were decreased in gastric cancer tissues, and the levels of methylamines (methylamine, DMA, TMAO) were obviously increased. Large levels of choline uptake and de novo synthesis are necessary for new membrane synthesis and one-carbon balance. Aberrations in choline metabolism have been demonstrated in a variety of cancers, including breast cancer [49] and colorectal cancer [18]. As shown in Table 2, the level of choline was down-regulated in gastric cancer tissues. The possible causes were as follows: first, dietary deficiency may affect the intake of choline because of the damage of the stomach. Second, the choline metabolism may be activated. Methylamines (methylamine, DMA, TMAO), products of choline metabolism, were elevated in gastric cancer tissues. Methylamines are usually regarded as nontoxic substances, which could induce hepatocarcinogenesis in rats [50]. So the similar mechanism may exist in human. Therefore, methylamines may indicate the disturbance of liver homeostasis in development of gastric cancer.

## Conclusions

In summary, utilizing the ^1^H NMR spectroscopy combined with multivariate statistical analysis, we identified significant metabolic shifts between gastric cancer tissues and normal controls. 48 distinguishing metabolites were identified, which constructed a diagnostic model for gastric cancer with a high area under the curve value. Moreover, we firstly identified the metabolic profile between the various stages cancer subjects and normal controls. A panel of 13 metabolites was changed along with the procession of gastric cancer, which may be related to the occurrence and even development of cancer. On the basis of this research, we believed that the metabolic information obtained by ^1^H NMR might play a significant role in screening biomarkers and the early diagnosis of gastric cancer. Further functional and clinical sample analysis of these distinguishing metabolites is needed to demonstrate the potential utility and the related mechanism underlying the gastric cancer.

### Ethics approval and consent to participate

The study was approved by the Ethics Committee of West China Hospital of Sichuan University and was also in accordance with the Declaration of Helsinki in 1975. The tissue samples used in this study have been collected in West China Hospital of Sichuan University. We obtained written informed consent from all the participants prior to the study. No financial incentive was provided to the participants and all human tissues were processed anonymously.

### Consent for publication

Not applicable.

### Availability of data and materials

The primitive NMR data are delivered as Additional file 6, and data are analysed by MestReNova software. The metabolines are identified by the related articles [18, 21–23] and the Human Metabolome (Database http://www.hmdb.ca/).

## Additional files

Additional file 1: Figure S1. PCA scores plot between the gastric cancer tissues and normal controls using ^1^H NMR. Black triangles represent normal controls, red blocks represent stage I of gastric cancer tissues, blue blocks represent stage II, green blocks represent stage III, yellow blocks represent stage IV. (JPG 54 kb) Additional file 2: Figure S2. OPLS-DA scores plot among different stage-related gastric cancer tissues. Red blocks represent stage I of gastric cancer tissues, blue blocks represent stage II, green blocks represent stage III, yellow blocks represent stage IV. (JPG 140 kb) Additional file 3: Figure S3. Three-dimensional OPLS-DA score plot among different stage-related gastric cancer tissues. Red blocks represent stage I of gastric cancer tissues, blue blocks represent stage II, green blocks represent stage III, yellow blocks represent stage IV. (JPG 156 kb) Additional file 4: Table S1. Metabolite changes between each stage of gastric cancer patients and normal controls. (DOCX 34 kb) Additional file 5: Figure S4. The histograms illustrating discrimination between gastric cancer tissues and normal controls corresponding to the constructed metabolic pathway map. Bar chart left to right: normal controls (blue bar), gastric cancer (red bar); the *Y* axis represents relative abundance of NMR signals (normalized to the total peaks). *, *p <* 0.05; **, *p <* 0.01. (JPG 214 kb) Additional file 6: The primitive NMR results. (ZIP 467 mb)

## Abbreviations

AJCC: the American Joint Committee on Cancer
AUC: area under the curve
FDR: false- discovery rate
NMR: nuclear magnetic resonance
OPLS-DA: orthogonal partial least-squares discriminant analysis
PCA: principal component analysis
PLS-DA: partial least-squares discriminant analysis
ROC: receiver operating curve
VIP: variable importance in the projection
