# Supplementary figures and images for: Tissue metabolic profiling of human gastric cancer assessed by 1H NMR
Source: BMC Cancer. 2016 Jun 29;16:371. doi: 10.1186/s12885-016-2356-4 (PMC4928316; doi:10.1186/s12885-016-2356-4)

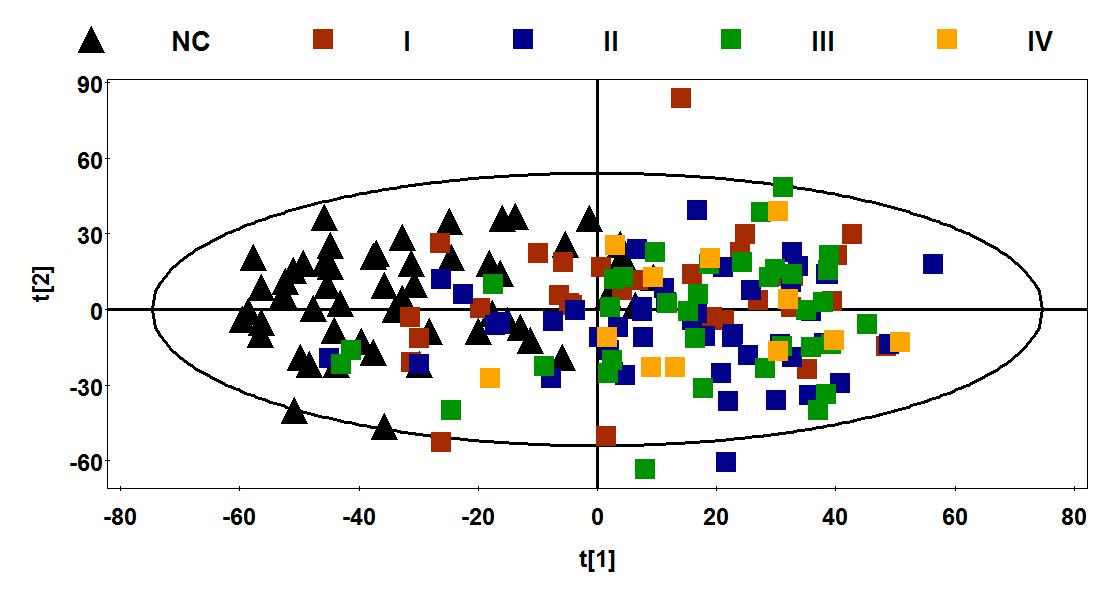

Supplement: Additional file 1: Figure S1. — PCA scores plot between the gastric cancer tissues and normal controls using 1H NMR. Black triangles represent normal controls, red blocks represent stage I of gastric cancer tissues, blue blocks represent stage II, green blocks represent stage III, yellow blocks represent stage IV. (JPG 54 kb) [file 12885_2016_2356_MOESM1_ESM.jpg]

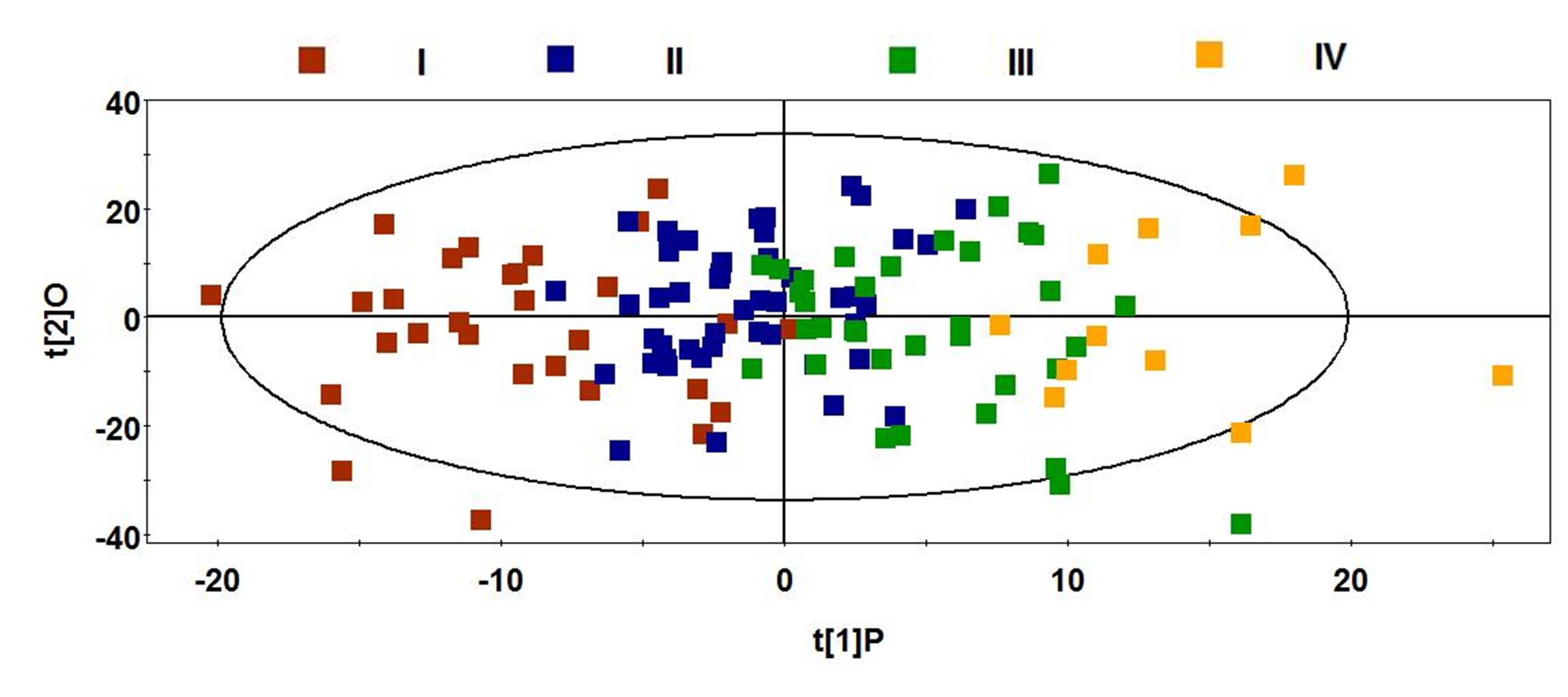

Supplement: Additional file 2: Figure S2. — OPLS-DA scores plot among different stage-related gastric cancer tissues. Red blocks represent stage I of gastric cancer tissues, blue blocks represent stage II, green blocks represent stage III, yellow blocks represent stage IV. (JPG 140 kb) [file 12885_2016_2356_MOESM2_ESM.jpg]

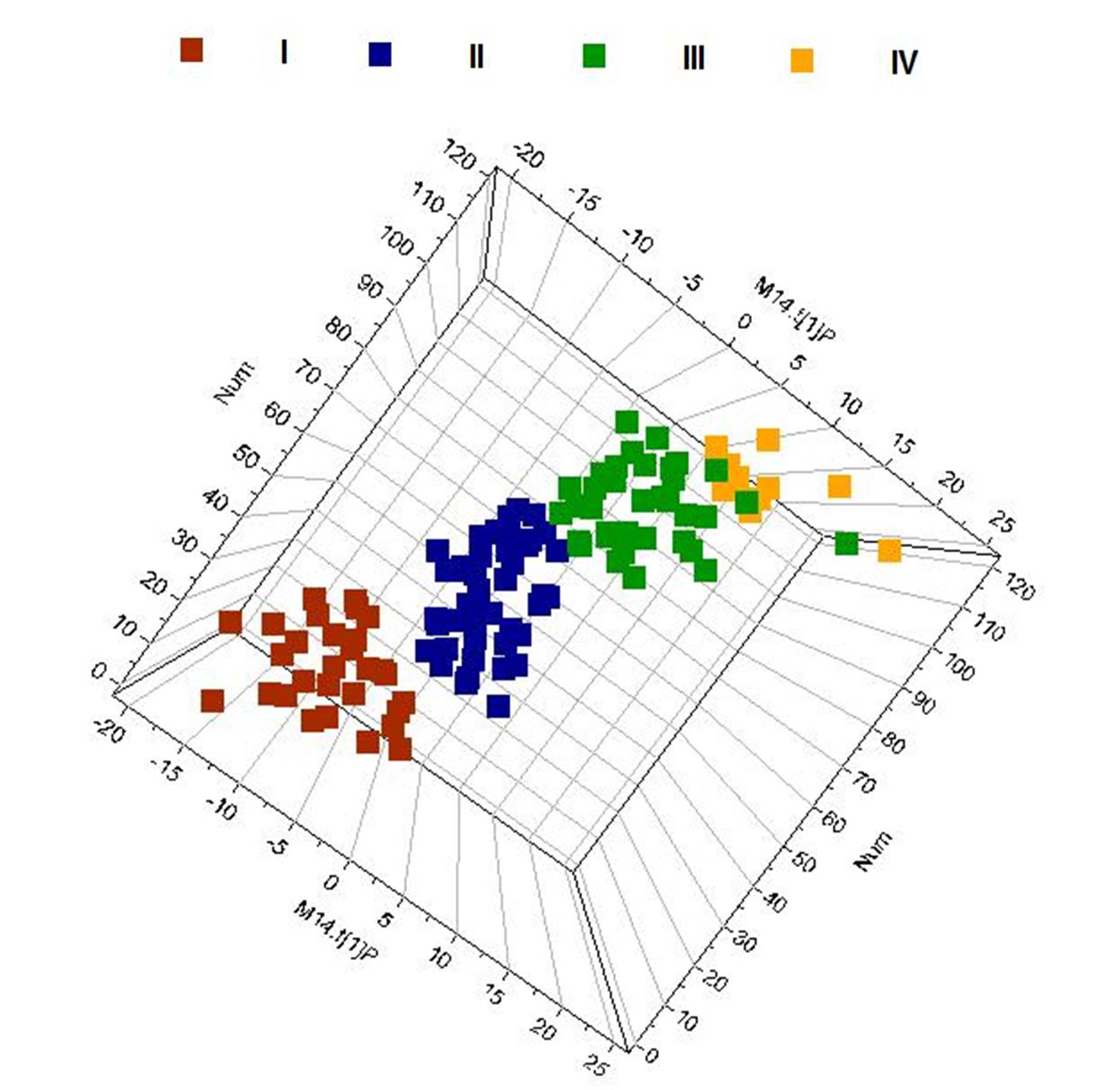

Supplement: Additional file 3: Figure S3. — Three-dimensional OPLS-DA score plot among different stage-related gastric cancer tissues. Red blocks represent stage I of gastric cancer tissues, blue blocks represent stage II, green blocks represent stage III, yellow blocks represent stage IV. (JPG 156 kb) [file 12885_2016_2356_MOESM3_ESM.jpg]

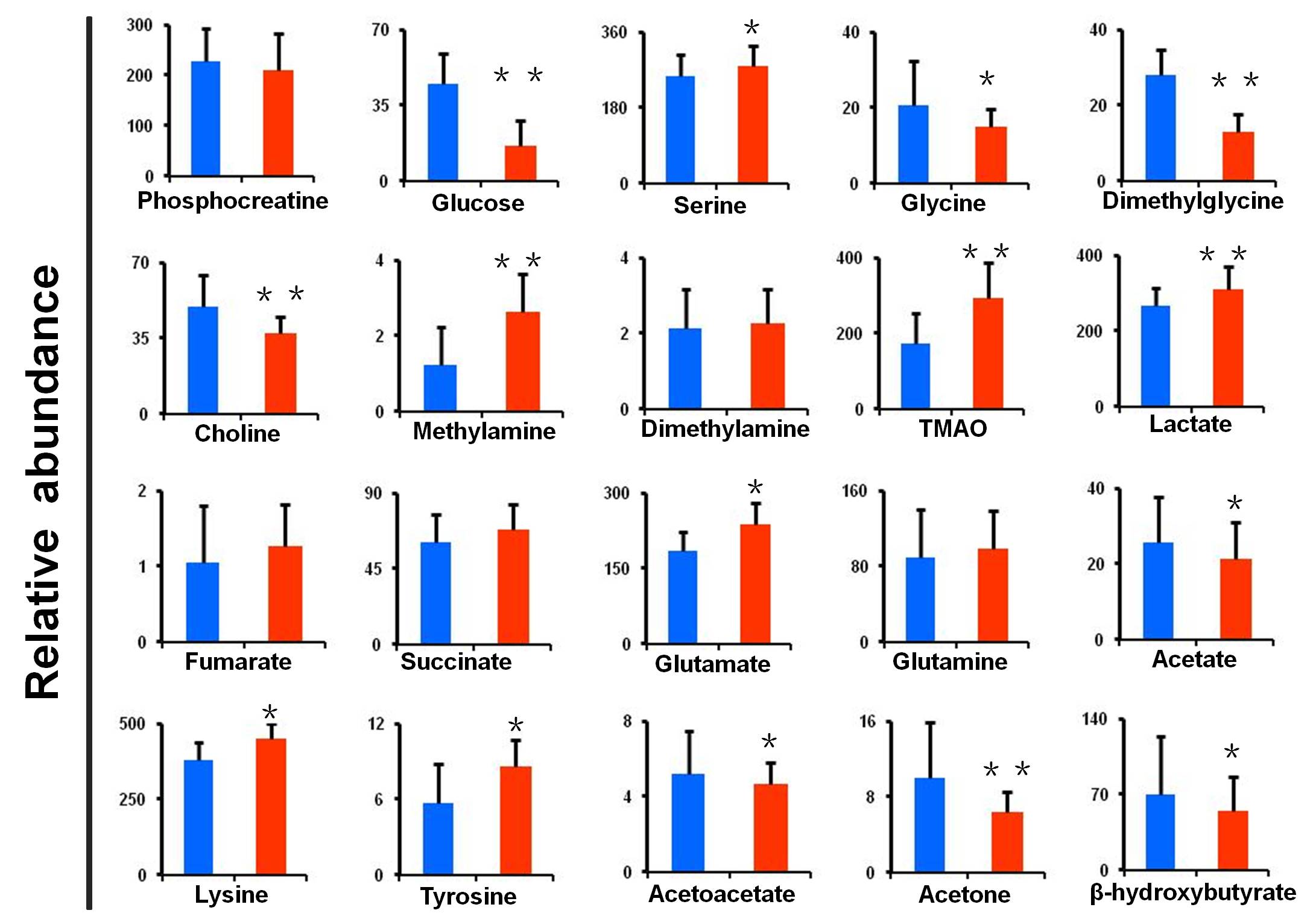

Supplement: Additional file 5: Figure S4. — The histograms illustrating discrimination between gastric cancer tissues and normal controls corresponding to the constructed metabolic pathway map. Bar chart left to right: normal controls (blue bar), gastric cancer (red bar); the Y axis represents relative abundance of NMR signals (normalized to the total peaks). *, p < 0.05; **, p < 0.01. (JPG 214 kb) [file 12885_2016_2356_MOESM5_ESM.jpg]
