# Supplementary material for: Tissue metabolic profiling of human gastric cancer assessed by 1H NMR
Source: BMC Cancer. 2016 Jun 29;16:371. doi: 10.1186/s12885-016-2356-4 (PMC4928316; doi:10.1186/s12885-016-2356-4)
Supplement: Additional file 4: Table S1. — Metabolite changes between each stage of gastric cancer patients and normal controls. (DOCX 34 kb) [file 12885_2016_2356_MOESM4_ESM.docx]

**Additional file 4.** Metabolite changes between each stage of gastric cancer patients and normal controls

| Metabolites | chemical shift | Mutiplicity^a^ | I vs Normal control | | |  | II vs Normal control | | |  | III vs Normal control | | |  | IV vs Normal control | | |
| --- | --- | --- | --- | --- | --- | --- | --- | --- | --- | --- | --- | --- | --- | --- | --- | --- | --- |
|  | （ppm） |  | VIP^b^ | *P*-Value^c^ | FC^d^ |  | VIP^b^ | *P*-Value^c^ | FC^d^ |  | VIP^b^ | *P*-Value^c^ | FC^d^ |  | VIP^b^ | *P*-Value^c^ | FC^d^ |
| VLDL: CH_3_-(CH_2)n-_ | 0.89 | br | 1.73 | <0.001 | -1.35 |  | 1.83 | <0.001 | -1.33 |  | 1.36 | 0.001 | -1.28 |  |  |  | -1.19 |
| Isoleucine | 0.94 | t | 1.09 | 0.023 | 1.04 |  | 1.86 | <0.001 | 1.14 |  | 2.18 | <0.001 | 1.14 |  | 2.09 | <0.001 | 1.17 |
|  | 1.01 | d | 2.61 | <0.001 | 1.27 |  | 2.82 | <0.001 | 1.40 |  | 2.89 | <0.001 | 1.40 |  | 2.86 | <0.001 | 1.40 |
| Leucine | 0.96 | t | 1.25 | <0.01 | -1.06 |  | 1.94 | <0.001 | 1.06 |  | 2.04 | <0.001 | 1.02 |  | 1.88 | <0.01 | 1.02 |
| Valine | 0.99 | d | 2.56 | <0.001 | 1.06 |  | 2.81 | <0.001 | 1.13 |  | 2.79 | <0.001 | 1.15 |  | 2.75 | <0.001 | 1.15 |
|  | 1.01 | d | 2.51 | <0.001 | 1.05 |  | 2.76 | <0.001 | 1.11 |  | 2.77 | <0.001 | 1.11 |  | 2.63 | <0.001 | 1.12 |
| β-hydroxybutyrate | 1.20 | d | 0.67 | 0.211 | -1.35 |  | 1.39 | <0.001 | -1.40 |  | 0.73 | 0.076 | -1.21 |  | 0.75 | 0.336 | -1.23 |
|  | 4.16 | d | 2.61 | <0.001 | 1.01 |  | 2.24 | <0.001 | 1.07 |  | 2.52 | <0.001 | -1.02 |  | 2.11 | <0.01 | -1.01 |
| Lactate | 1.33 | d | 2.35 | <0.01 | 1.16 |  | 3.09 | <0.001 | 1.23 |  | 2.94 | <0.001 | 1.26 |  | 3.35 | <0.01 | 1.24 |
|  | 4.11 | q |  |  | 1.08 |  | 2.08 | <0.001 | 1.18 |  | 1.52 | <0.001 | 1.17 |  | 2.39 | <0.001 | 1.21 |
| 2-Hydroxyisobutyric acid | 1.44 | s | 1.54 | 0.041 | -1.05 |  | 1.36 | <0.01 | -1.02 |  | 1.35 | 0.001 | -1.01 |  |  |  | -1.04 |
| Citrulline | 1.57 | m | 1.34 | <0.01 | -1.67 |  | 2.08 | <0.001 | -2.30 |  | 2.16 | <0.001 | -2.72 |  | 1.79 | <0.01 | -3.18 |
| VLDL: -CH_2_-CH_2_-CH_2_O | 1.58 | br | 1.76 | <0.001 | -1.71 |  | 2.17 | <0.001 | -1.79 |  | 2.03 | <0.001 | -1.67 |  | 2.01 | <0.01 | -2.21 |
| Acetate | 1.93 | s | 2.03 | <0.001 | -1.16 |  | 2.01 | <0.001 | -1.12 |  | 2.28 | <0.001 | -1.28 |  | 2.08 | <0.01 | -1.25 |
| N-Acetyl glycoprotein | 2.05 | s | 1.21 | 0.015 | 1.04 |  | 1.85 | <0.001 | 1.20 |  | 1.84 | <0.001 | 1.14 |  | 1.48 | 0.032 | 1.11 |
| O-Acetyl glycoprotein | 2.07 | s | 3.63 | <0.001 | 2.20 |  | 3.62 | <0.001 | 2.03 |  | 3.53 | <0.001 | 2.16 |  | 2.95 | <0.001 | 1.88 |
| D-ribose | 2.23 | s | 1.57 | <0.001 | -1.49 |  | 1.84 | <0.001 | -1.63 |  | 1.72 | <0.001 | -1.51 |  | 1.27 | 0.071 | -1.68 |
| Acetone | 2.23 | s | 1.57 | <0.001 | -1.49 |  | 1.84 | <0.001 | -1.63 |  | 1.72 | <0.001 | -1.51 |  | 1.27 | 0.071 | -1.68 |
| Lipid,-CH_2_-C=O | 2.26 | br | 1.36 | <0.01 | -1.16 |  | 1.82 | <0.001 | -1.25 |  | 1.64 | <0.001 | -1.16 |  | 1.32 | 0.047 | -1.09 |
| Acetoacetate | 2.28 | s |  |  | -1.20 |  |  |  | -1.24 |  | 1.18 | <0.01 | -1.08 |  | 1.81 | <0.01 | 1.03 |
| Acetoacetic acid | 2.31 | s | 1.37 | <0.01 | -1.33 |  | 1.56 | <0.001 | -1.40 |  | 1.20 | <0.01 | -1.20 |  | 1.16 | 0.117 | -1.19 |
| Glutamate | 2.36 | m | 2.96 | <0.001 | 1.28 |  | 2.54 | <0.001 | 1.25 |  | 2.77 | <0.001 | 1.35 |  | 3.17 | <0.001 | 1.24 |
|  | 3.77 | m | 2.03 | <0.001 | 1.16 |  | 2.50 | <0.001 | 1.18 |  | 2.33 | <0.001 | 1.19 |  | 2.87 | <0.001 | 1.18 |
| Succinate | 2.41 | s | 2.36 | <0.001 | 1.01 |  | 1.86 | <0.001 | 1.03 |  | 2.55 | <0.001 | 1.01 |  | 2.41 | <0.001 | 1.08 |
| Glutamine | 2.46 | m | 2.60 | <0.001 | 1.05 |  | 2.06 | <0.001 | -1.01 |  | 2.46 | <0.001 | 1.06 |  | 1.94 | <0.01 | 1.01 |
| Glutathione | 2.56 | m | 2.74 | <0.001 | 1.51 |  | 2.44 | <0.001 | 1.51 |  | 2.61 | <0.001 | 1.55 |  | 3.11 | <0.001 | 1.40 |
|  | 2.96 | m | 2.52 | <0.001 | 1.40 |  | 2.15 | <0.001 | 1.42 |  | 2.54 | <0.001 | 1.55 |  | 2.81 | <0.001 | 1.39 |
| Methylamine | 2.59 | s | 2.48 | <0.001 | 2.32 |  | 2.35 | <0.001 | 2.32 |  | 2.28 | <0.001 | 2.30 |  | 2.10 | <0.01 | 1.66 |
| Aspartate | 2.68 | dd | 1.86 | <0.001 | 1.59 |  | 1.89 | <0.001 | 1.52 |  | 1.79 | <0.001 | 1.58 |  | 2.03 | <0.01 | 1.40 |
|  | 2.82 | dd | 2.29 | <0.001 | 1.75 |  | 2.13 | <0.001 | 1.51 |  | 2.46 | <0.001 | 1.54 |  | 2.76 | <0.001 | 1.29 |
| Dimethylamine | 2.73 | s | 1.43 | <0.01 | 1.04 |  | 1.33 | <0.01 | 1.09 |  | 1.74 | 0.071 | 1.11 |  | 1.00 | 0.127 | 1.29 |
| Acetic acid | 3.00 | s |  |  | -1.07 |  | 1.92 | <0.001 | -1.12 |  | 1.74 | <0.001 | -1.08 |  | 1.62 | 0.019 | -1.09 |
| Phosphocreatine | 3.04 | s | 1.26 | 0.013 | 1.01 |  | 1.67 | <0.001 | -1.13 |  | 1.51 | <0.001 | -1.09 |  | 1.56 | 0.016 | -1.14 |
|  | 3.93 | s | 1.63 | <0.01 | -1.01 |  | 1.23 | <0.01 | -1.12 |  | 0.98 | 0.024 | -1.10 |  | 1.19 | 0.098 | -1.09 |
| Creatine | 3.04 | s | 1.26 | <0.01 | 1.01 |  | 1.67 | <0.001 | -1.13 |  | 1.51 | <0.001 | -1.09 |  | 1.56 | 0.016 | -1.14 |
|  | 3.94 | s | 1.63 | <0.01 | -1.01 |  | 1.23 | <0.01 | -1.12 |  | 0.98 | 0.024 | -1.10 |  | 1.19 | 0.098 | -1.09 |
| Creatinine | 3.04 | s | 1.26 | <0.01 | 1.01 |  | 1.67 | <0.001 | -1.13 |  | 1.51 | <0.001 | -1.09 |  | 1.56 | 0.016 | -1.14 |
|  | 3.45 | s | 1.30 | <0.01 | -1.08 |  | 1.64 | 0.187 | 1.05 |  | 1.18 | 0.01 | -1.06 |  | 1.05 |  | 1.15 |
| Choline | 3.20 | s | 1.94 | 0.052 | -1.28 |  | 1.80 | 0.039 | -1.25 |  | 1.03 | 0.016 | -1.56 |  | 1.84 | 0.212 | -1.29 |
| PC（phosphochline） | 3.21 | s | 2.17 | <0.001 | -1.42 |  | 1.39 | <0.01 | -1.32 |  | 1.75 | <0.001 | -1.49 |  | 1.74 | <0.01 | -1.23 |
| Trimethylamine-N-oxide(TMAO) | 3.27 | s | 1.64 | <0.001 | 1.59 |  | 1.65 | <0.001 | 1.73 |  | 1.97 | <0.001 | 1.82 |  | 2.10 | <0.01 | 1.57 |
| myo-Inositol | 3.55 | dd | 2.46 | <0.001 | -1.46 |  | 2.68 | <0.001 | -1.90 |  | 2.13 | <0.001 | -1.63 |  | 2.72 | <0.001 | -1.72 |
|  | 3.63 | t | 1.68 | <0.01 | -1.27 |  | 2.85 | <0.001 | -1.55 |  | 2.21 | <0.001 | -1.40 |  | 2.51 | <0.001 | -1.50 |
|  | 4.06 | m | 1.70 | <0.01 | -1.35 |  | 2.82 | <0.001 | -1.64 |  | 2.21 | <0.001 | -1.43 |  | 2.54 | <0.001 | -1.54 |
| α-Glucose | 3.55 | dd | 2.46 | <0.001 | -1.46 |  | 2.68 | <0.001 | -1.90 |  | 2.13 | <0.001 | -1.63 |  | 2.72 | <0.001 | -1.72 |
|  | 5.23 | d | 2.74 | <0.001 | -2.88 |  | 2.89 | <0.001 | -2.94 |  | 2.70 | <0.001 | -2.68 |  | 2.64 | <0.001 | -2.73 |
| Glycine | 3.57 | s | 1.23 |  | -1.46 |  | 1.91 | 0.024 | -1.20 |  | 1.13 | <0.01 | -1.40 |  | 1.83 | 0.197 | -1.42 |
| Glycerol | 3.64 | dd |  |  | -1.42 |  | 2.53 | <0.001 | -1.54 |  | 2.14 | <0.001 | -1.56 |  | 2.17 | <0.01 | -1.49 |
| Dimethylglycine | 3.71 | s | 2.58 | <0.001 | -2.19 |  | 2.53 | <0.001 | -2.07 |  | 2.70 | <0.001 | -2.36 |  | 2.54 | <0.001 | -2.10 |
| Lysine | 3.77 | m | 2.03 | <0.001 | 1.16 |  | 2.50 | <0.001 | 1.19 |  | 2.33 | <0.001 | 1.19 |  | 2.87 | <0.001 | 1.18 |
| Glycolate | 3.93 | s | 1.63 | <0.01 | -1.01 |  | 1.23 | <0.01 | -1.12 |  | 0.98 | 0.024 | -1.10 |  | 1.19 | 0.098 | -1.09 |
| Serine | 3.98 | m | 2.43 | <0.001 | 1.14 |  | 2.18 | <0.001 | 1.10 |  | 2.32 | <0.001 | 1.07 |  | 2.63 | <0.001 | 1.08 |
| Uracil | 5.80 | d | 2.42 | <0.001 | 4.30 |  | 2.60 | <0.001 | 5.67 |  | 2.33 | <0.001 | 4.48 |  | 2.67 | <0.001 | 4.46 |
|  | 7.54 | d | 2.60 | <0.001 | 2.13 |  | 2.87 | <0.001 | 2.61 |  | 2.57 | <0.001 | 2.19 |  | 2.79 | <0.001 | 2.16 |
| Fumarate | 6.53 | s | 1.33 | <0.01 | 1.11 |  | 1.01 | <0.01 | 1.24 |  | 1.12 | <0.01 | 1.14 |  | 1.47 | <0.01 | 1.12 |
| 4-hydroxyphenylactate | 6.88 | d | 1.78 | <0.001 | 1.35 |  | 1.95 | <0.001 | 1.62 |  | 2.17 | <0.001 | 1.51 |  | 2.88 | <0.001 | 1.51 |
|  | 7.18 | d | 1.85 | <0.001 | 1.15 |  | 2.05 | <0.001 | 1.28 |  | 2.47 | <0.001 | 1.20 |  | 2.78 | <0.001 | 1.24 |
| Tyrosine | 6.90 | d | 1.78 | <0.001 | 1.37 |  | 1.95 | <0.001 | 1.64 |  | 2.17 | <0.001 | 1.52 |  | 2.88 | <0.001 | 1.49 |
|  | 7.20 | d | 1.85 | <0.001 | 1.14 |  | 2.05 | <0.001 | 1.29 |  | 2.47 | <0.001 | 1.20 |  | 2.78 | <0.001 | 1.21 |
| Trytophan | 7.29 | m | 1.03 | <0.01 | -1.46 |  | 1.53 | <0.001 | -1.44 |  | 1.53 | <0.001 | -1.32 |  | 1.16 | 0.125 | -1.22 |
| Phenyacetylglutamine | 7.42 | m | 1.70 | <0.01 | 1.05 |  | 2.11 | <0.001 | 1.21 |  | 2.41 | <0.001 | 1.12 |  | 2.44 | <0.001 | 1.12 |
| Adenine | 8.12 | m |  |  | 1.23 |  | 1.32 | <0.01 | 1.24 |  | 1.31 | <0.01 | 1.21 |  | 1.09 | 0.101 | 1.03 |
| Hypoxanthine | 8.18 | s |  |  | -1.08 |  | 1.00 | 0.013 | -1.08 |  | 1.24 | <0.01 | -1.22 |  | 1.86 | <0.01 | -1.23 |
|  | 8.21 | s | 1.04 | 0.027 | -1.59 |  | 1.35 | <0.01 | -1.24 |  | 1.19 | <0.01 | -1.51 |  |  |  | -1.65 |
| Formate | 8.45 | s |  |  | 1.11 |  | 1.27 | <0.01 | -1.06 |  |  |  | -1.10 |  | 1.57 | 0.011 | -1.30 |

aMultiplicity: s, singlet; d, doublet; t, triplet; q, quartet; dd, doublet of doublets; m, multiplet; br, broad;

bVariable importance in the projection was obtained from OPLS-DA model with a threshold of 1.0.

cP-value obtained from Student's t-test.

dFold change(FC) between gastric cancer patients and normal controls. Fold change with a positive value indicates a relatively higher concentration present in gastric cancer patients while a negative value means a relatively lower concentration as compared to the normal controls.
